# Unravelling the ultrafast charge dynamics in PbS quantum dots through resonant Auger mapping of the sulfur K-edge†

**DOI:** 10.1039/d2ra06091d

**Published:** 2022-11-04

**Authors:** Tamara Sloboda, Fredrik O. L. Johansson, Birgit Kammlander, Elin Berggren, Sebastian Svanström, Alberto García Fernández, Andreas Lindblad, Ute B. Cappel

**Affiliations:** Division of Applied Physical Chemistry, Department of Chemistry, KTH Royal Institute of Technology SE-100 44 Stockholm Sweden fjson@kth.se; Sorbonne Université, CNRS, Institut des NanoSciences de Paris, INSP F-75005 Paris France; Division of X-ray Photon Science, Department of Physics and Astronomy, Uppsala University Box 516 751 20 Uppsala Sweden

## Abstract

There is a great fundamental interest in charge dynamics of PbS quantum dots, as they are promising for application in photovoltaics and other optoelectronic devices. The ultrafast charge transport is intriguing, offering insight into the mechanism of electron tunneling processes within the material. In this study, we investigated the charge transfer times of PbS quantum dots of different sizes and non-quantized PbS reference materials by comparing the propensity of localized or delocalized decays of sulfur 1s core hole states excited by X-rays. We show that charge transfer times in PbS quantum dots decrease with excitation energy and are similar at high excitation energy for quantum dots and non-quantized PbS. However, at low excitation energies a distinct difference in charge transfer time is observed with the fastest charge transfer in non-quantized PbS and the slowest in the smallest quantum dots. Our observations can be explained by iodide ligands on the quantum dots creating a barrier for charge transfer, which reduces the probability of interparticle transfer at low excitation energies. The probability of intraparticle charge transfer is limited by the density of available states which we describe according to a wave function in a quantum well model. The stronger quantum confinement effect in smaller PbS quantum dots is manifested as longer charge transfer times relative to the larger quantum dots at low excitation energies.

## Abstract 2

By measuring the resonant S-KLL Auger decay we investigate the attosecond charge transfer in PbS quantum dots of different sizes. The results show both intra- and interparticle charge transfer, where the former shows a strong QD size dependence.

## Graphical abstract

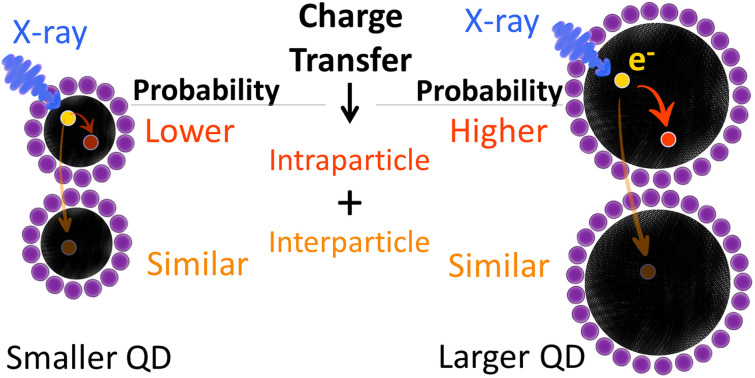

## Introduction

Quantum dots (QD) are a well-known type of semiconductor material often used in fields where precise control of the light emitted or absorbed by them is necessary. The most common applications include LEDs, biosensors, detectors, transistors, imaging and photovoltaic devices such as solar cells.^1–5^ Besides low-cost and relatively simple manufacturing methods quantum dots allow bandgap tuning through the size of the dot. This size-bandgap relation is a consequence of quantum confinement, first described in the 1980s by the group of L. Brus.^6,7^ For solar cells of the third generation, this has proven to be an exceptional quality since using different nanocrystal sizes and thus different bandgaps, the material may be tuned to absorb a specific range of electromagnetic spectrum.

One of the currently most studied quantum dot solar cell (QDSC) material are halide capped lead sulfide (PbS) quantum dots with roughly 1.3 eV bandgap and devices using these QDs have reached power conversion efficiency of above 13%,^8,9^ and the stability in air is reported to be over 300 h.^10^ Still, greater improvements are necessary to reach industrial standards. In attempt to contribute to the improvements, many studies are done on the charge generation, charge transport from QDs to the electrodes of the cell and charge dynamics in general, using time-resolved pump-probe methods.^11–18^ In these methods, the excitation of the particle usually happens from the valence bands, which are delocalized all over the particle. Consequently, it is difficult to determine which atoms are being excited, and if they are in any way affected by the presence of the created vacancies. In the present study, we investigated X-ray core-excited states that decay through auto-ionization. In contrast with the common pump-probe methods, when using core-excitations from deep core levels there is very little to no overlap with the valence band, and the influence of the present vacancies is diminished.

As a first step, we determined the resonant region of PbS material by exciting the S K absorption edge (to S p states) which allowed us to study the unoccupied states in the PbS samples using X-ray absorption spectroscopy (XAS). In this excitation energy range, around the S K edge, mapping of the S KL_2,3_L_2,3_ Auger electron energy range reveal the dynamics of this excited in resonant Auger spectroscopy (RAS). Finally, by applying RAS in core hole clock spectroscopy (CHCS) using the lifetime of the core hole (created by the resonant excitation) as an internal clock^19^ it is possible determine the charge transfer (CT) times in the quantum dot PbS and the continuous PbS film samples. This initial charge transfer is localized and unaffected by holes in the valence band. Herein, we present the impact that the quantum dot size *i.e.* quantum confinement has on the ultrafast (sub-femtosecond) charge transfer dynamics in PbS materials. Few studies have been performed using CHCS to study photovoltaic materials^20–23^ and our study concerns PbS quantum dots in comparison to PbS in thin films.

## Experimental details

All chemicals in the synthesis were purchased from Sigma-Aldrich except for the PbI_2_ and PbBr_2_, which were ordered from TCI.

The PbS colloidal QDs are synthesized as previously reported^24^ with several modifications depending on the nanocrystal size, with main parameters summarized in Table 1. The lead oxide (PbO, 99.99%), oleic acid (OA, tech. grade 90%) and 1-octadecene (ODE, tech. grade 90%) were added to a round bottom flask (in ratios according to Table 1) and the mixture is first degassed under vacuum for roughly 2 h while heated to about 110 °C. Before the injection of hot hexamethyldisilathiane solution in 8–10 ml ODE (TMS, about 80 °C), the mixture was introduced to nitrogen atmosphere and stirred until the temperature stabilized at injection temperature specified in Table 1. The hot TMS was then quickly injected, giving rise to an instantaneous reaction, visible through color change of the reaction mixture from transparent to very dark brown. The heating source is removed 2–3 minutes after the injection, letting the PbS–OA particles cool down to room temperature. The newly formed PbS–OA quantum dots were cleaned in two washing steps with acetone and toluene, dried under vacuum and finally dispersed in octane so that the concentration was 50 mg ml^−1^.

**Table 1:** PbS quantum dots synthesis parameters

Aimed QD size	2 nm		3 nm		5 nm	
Reagent	[mmol]		[mmol]		[mmol]	
PbO	4	0.93 g	4	0.93 g	2	0.45 g
TMS	2	0.42 ml	2	0.42 ml	1	0.21 ml
OA	7	2.2 ml	14	4.53 ml	28	8.84 ml
ODE		25 ml		25 ml		20 ml
Injection temperature	70 °C		90 °C		100 °C	

The PbS reference thin film was prepared as previously reported,^24^ while the natural galena crystal was ordered from Crystal Cave Rocks shop and cleaved in air just before being stored into vacuum chamber.

Details on the sample preparation and spin-coating of quantum dot films can be found in the ESI.†

Hard X-ray Photoelectron Spectroscopy (HAXPES), resonant Auger spectroscopy (RAS) and X-ray absorption (XAS) measurements were carried out at the HIKE endstation^25^ at the KMC-1 beamline^26^ at the BESSY II electron storage ring operated by the Helmholtz-Zentrum Berlin für Materialien und Energie, where the available photon energies range from 2 keV to 12 keV. The photon energy was selected using a Si double crystal monochromator and the X-rays were focused on the sample using a parabolic glass capillary. The pressure in the analysis chamber was low 10^−8^ mbar and a VG Scienta R4000 hemispherical electron energy analyzer was used to measure the kinetic energies of the electrons emitted from the sample. The binding energy scale was calibrated by measuring the Au 4f_7/2_ core level of a gold foil mounted on the manipulator and setting its position to 84.0 eV binding energy, while the photon energy scale for the XAS data was calibrated using the first and third order X-rays from the monochromator and measuring Au 4f photoelectron spectra. The XAS data was collected using a Bruker XFlash 4010 fluorescence detector mounted on the same experiment chamber. All of the measurements were always carried out on fresh sample spots.

## Results and discussions

**Fig. 1:**
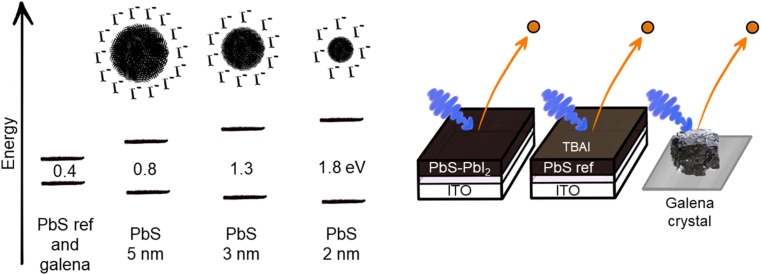
Illustration of the bandgap values according to absorption measurements of PbS quantum dots of different size and sample architecture of PbS QD sample, PbS ref continuous thin film sample (with tetrabutylammonium iodide (TBAI) surface treatment) and galena crystal.

The samples used in this experiment are continuous PbS thin film (PbS ref) prepared as previously reported,^24^ quantum dot samples of three different sizes, with a PbI_2_ surface treatment, and a cleaved natural galena PbS crystal (galena), the sample architecture is presented in Fig. 1.

Oleic acid-capped PbS colloidal quantum dot solutions were characterized using UV-Vis-NIR absorption and photoluminescence (Fig. S1†), prior to ligand exchange and film deposition. The absorption and emission peaks of the quantum dots show smaller Stokes shift as the diameter of the dot increases. This has been previously observed in lead sulfide quantum dots^17,27^ and has been assigned to existence of electron states within the bandgap, which could be surface trap states or defects.^28,29^ In the absorption spectra the QDs showed clear differences in exciton peak positions 1.79 eV, 1.29 eV and 0.81 eV center energy, as obtained from a least squares curve fitting with a Gaussian distribution (see Table S1, and Fig. 1 and S1†). According to the bandgap-size equation reported by Moreels *et al.*^30^ these bandgaps come from quantum dots with diameters 2.15 nm, 3.14 nm and 5.82 nm. Using the width of the absorption peak to estimate size distributions, we find that these were in the range of 2.0–2.3 nm, 2.9–3.3 nm and 5.6–6.1 nm, respectively (Table S1†). This suggests that the size distribution is similar for the quantum dots of different dot sizes. Moreover, the rock salt structure of the PbS QDs was confirmed (Fig. S1†) using X-ray diffraction (XRD) of solid films of drop-casted colloidal PbS–OA. We confirmed the sizes of the particles using the Scherrer equation,^31^ showing that the results from the two methods are in good agreement (Table S1†). Therefore, we label the samples as PbS 2 nm, PbS 3 nm and PbS 5 nm in the rest of the paper.

**Fig. 2:**
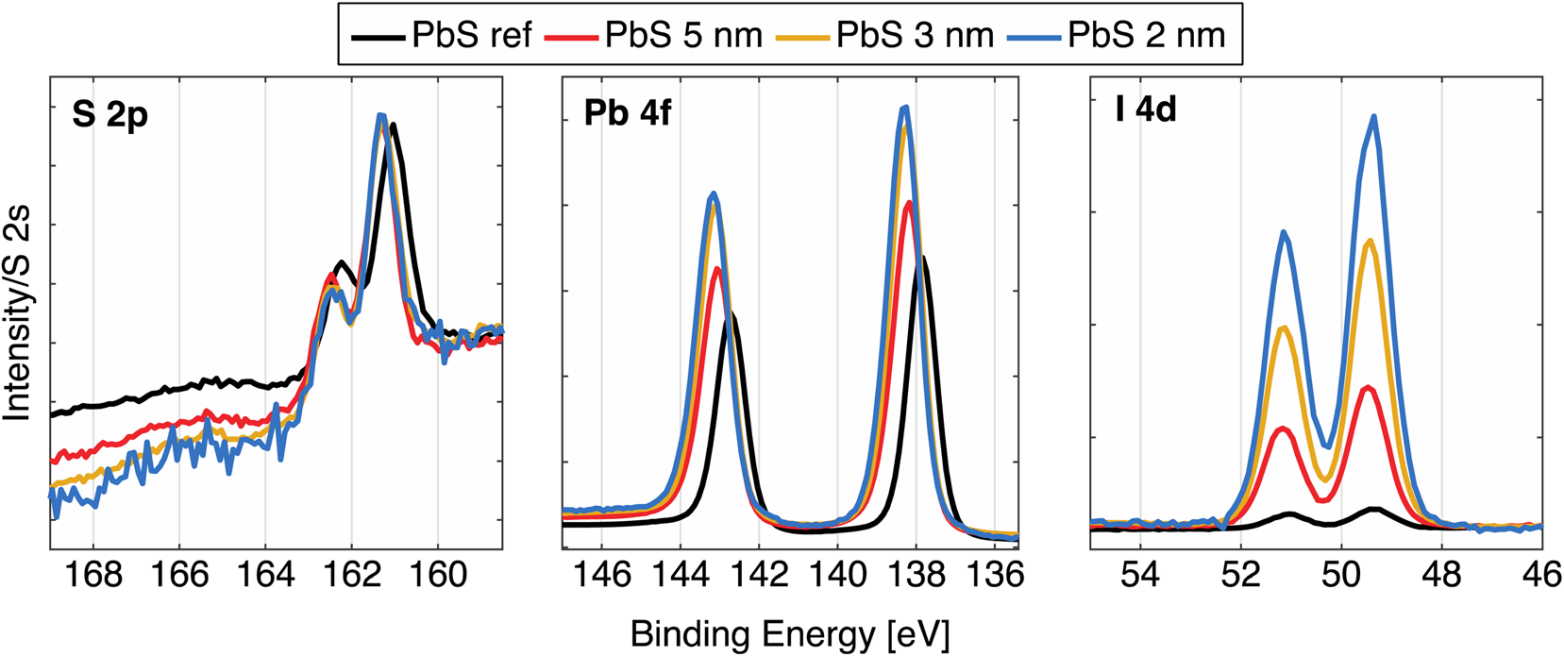
High resolution HAXPES spectra of the PbS samples: S 2p, Pb 4f, and I 4d, calibrated against an external gold reference mounted on the manipulator, and normalized to the S 2s intensity.

After ligand exchange from oleic acid to iodide ligands, the samples were further characterized using hard X-ray photoelectron spectroscopy (HAXPES) using a photon energy of 3000 eV. All the core levels from PbS QD samples are shifted to higher BE in relation to the core levels from PbS reference sample (Fig. 2 and Table S2†), which matches results in our previously reported study.^24^ In case of the cleaved galena crystal, the core levels were slightly shifted to lower binding energy relative to the PbS reference sample (Fig. S2, Table S2†). Galena also exhibits additional Pb contributions in the Pb 4f spectra compared to PbS ref (Fig. S2†) implying the presence of impurities in the crystal, possibly due to imperfect cleaving the crystal (in ambient atmosphere). In comparison of the PbS QD and PbS ref, the shapes of Pb 4f core level spectra shown in Fig. 2 are similar. However, the relative ratio of S 2p and I 4d with respect to the Pb 4f contribution varies between the four samples (Fig. S3†). Specifically, the PbS ref shows more sulfur and less iodine (due to the surface treatment) in relation to lead when compared to the QDs. The PbS 2 nm and PbS 3 nm show even lower sulfur and higher iodine content compared to the 5 nm sample.

The fact there is more iodide in the smaller nanocrystals is expected as a consequence of the high surface/volume ratio in small PbS particles.^32^ In fact, thinking of these ions as spheres and calculating the surface/volume ratio in terms of simplest geometry, the surface/volume ratio for PbS 2 nm, PbS 3 nm and PbS 5 nm becomes 3, 2 and 1.2 nm^−1^, respectively, while the volume ratio of the surface layer consisting of iodide ions compared to the PbS volume in quantum dot is 0.8, 0.5 and 0.3. Calculation details may be found in the ESI.† From the HAXPES intensity ratios (Table S2†), we obtain ratios of the areas of I 4d and Pb 4f spectral features to be 0.65, 0.46 and 0.29, which conforms to the trend of iodide/lead ratios as a function of size estimated by this simplified model. In addition to this, the sulfur to lead ratio implies that ratio of surface Pb ions/bulk Pb ions is greater for smaller dots than in the larger ones. The surface quality of the thin films can be estimated by looking at S 1s spectra at this photon energy, since the low kinetic energy of these electrons (around 530 eV) results in a short inelastic mean free path and hence a more surface sensitive measurement (91% of the signal comes from the first 3 nm of the sample^24^). As shown in Fig. S4,† the largest S 1s contribution comes from the sulfur in the quantum dots (S bonded to Pb), with traces of S–C, S–H and S–O contributions, suggesting that surface of all PbS samples is neither contaminated nor degraded to a significant extent.^24^ The S 1s of the natural galena also shows a clean surface with small additional contributions to the S 1s spectrum (Fig. S2 and S4†), which makes this sample comparable to other PbS samples in this study. In addition to this, we observed signals from C 1s and O 1s, similar to our previous studies.^24,33^ The galena crystal showed a larger oxygen contribution, which possibly comes from lead oxide compounds (Fig. S2 and S4†), while the amount of carbon is similar to the rest of PbS samples.

### Valence band and unoccupied states

As mentioned before, the size of the quantum dot affects the bandgap of the material, which is expected to be reflected in the valence and conduction band spectra. The left side of Fig. 2 shows the valence band spectra of the PbS reference and the QD samples. While all valence band (VB) spectra of the quantum dot samples look similar, the largest difference is observed in the position of the VB onset of the PbS reference sample (0.27 eV) in comparison to VB onsets of QD samples which are shifted to higher BE (Table S2†). In addition to this, a small shift in the VB onset towards higher binding energy with the QD diameter increase was observed. A similar small shift was observed earlier in a study of Miller *et al.* where it is explained as a consequence of the fact that the valence band maximum (VBM) states (due to lower population of states corresponding to the S 3p states) are not necessarily found at the same position as the fitted onset of the VB spectra.^34^ From the photoelectron spectroscopy measurements of quantum dots of various sizes and application of density of states (DOS) model, the authors propose the following correction for the valence band maximum position:

1 VBM = VB_onset_ − 0.382 + 0.226*E*_g_

where *E*_g_ is the bandgap of the quantum dot. The VBM calculated using this correction gives VB values which reflect the size difference more clearly (Table S2†).

**Fig. 3:**
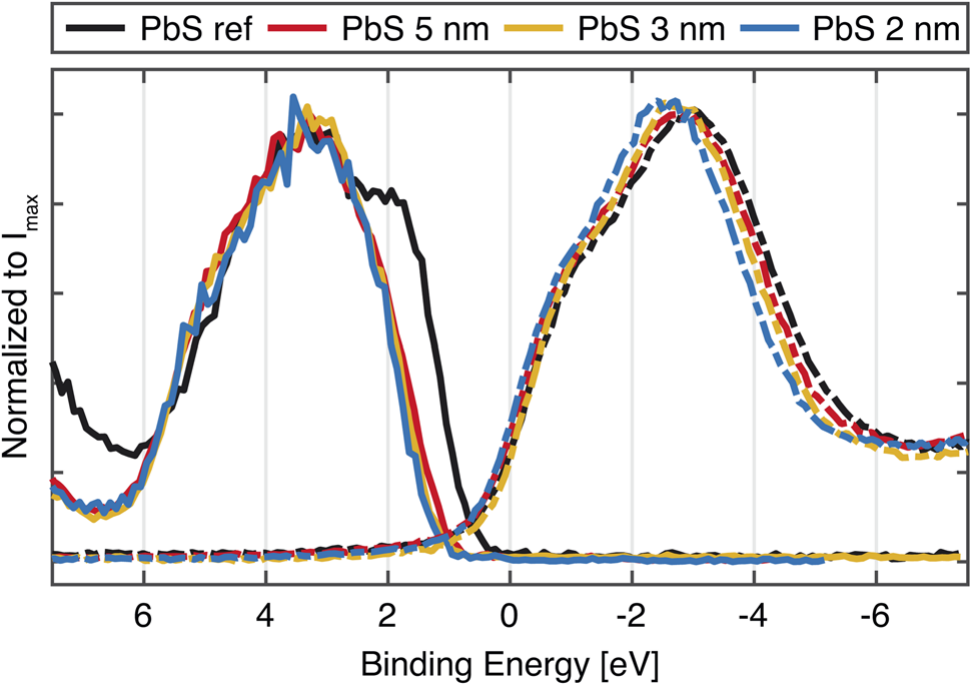
Valence band PES spectra and XAS spectra of PbS 2 nm, PbS 3 nm, PbS 5 nm and PbS ref. The PES spectra were recorded with a photon energy of 3000 eV and are calibrated against external gold reference and normalized to the intensity maximum. The XAS spectra are also normalized to the intensity maximum, while the energy axis is calculated from the difference between the photon energy and the S 1s core level position.

The unoccupied states were investigated through measurements of the sulfur K-edge X-ray absorption (XAS) spectra, which selectively probes the S 3p orbitals. In order to be plotted together with the VB spectra (Fig. 3, right), the energy scale of the original spectra (Fig. S5†) was converted to a binding energy scale from the difference of the photon energy axis of the absorption spectra and binding energy (BE) position of the S 1s core level. The XAS spectra show similar positions of the absorption features, with only small differences between the QDs and the PbS ref. All of the samples show characteristic contributions from mixed S 3p and Pb 6s orbitals (marked with 1 and 2 in Fig. S5†) and the contributions attributed to the scattering effects due to geometry of Pb and S atoms in PbS crystal (3 and 4 in Fig. S5†), which have been observed previously.^35,36^ In the study of Trejo *et al.*, the lack of feature shifting upon a size reduction of the PbS was explained as the confirmation that the oxidation state of the sulfur remains unchanged,^36^ as the shifts coming from a oxidation state change are known to significant.^37^ This is reflected in our data as well, although a small shift of the absorption maximum towards more negative values is visible with decreasing quantum confinement. The width of the absorption feature is narrower for the smallest QDs and wider for the PbS reference (Fig. 2), which could reflect the narrowing of bands due to quantum confinement. However, no significant changes in the position of the absorption edge are observed, which could be assigned to the change in conduction band position with quantum dot size. In the previous study by Miller *et al.*,^34^ it was also found that the conduction band positions determined by inverse photoelectron spectroscopy did not vary with the PbS quantum dot size as expected. The suggested explanation for this was that the density of states near the conduction band edge is low for PbS with a small band gap and the conduction band onset is therefore not accurately observed. This could be also the case here and the X-ray absorption spectra can therefore not be used to give precise estimates of the conduction band edge. Instead, they highlight the main density of states with S 3p character in the conduction band.

### Core hole clock spectroscopy

After mapping out the X-ray absorption upon excitation from S 1s unoccupied states, we investigated the S KL_2,3_L_2,3_ Auger signal in resonant conditions at photon energies ranging from 2460 to 2500 eV. Core hole clock spectroscopy (CHCS)^19^ is an application of Resonant Auger spectroscopy (RAS) for determining electron charge transfer times on the femto- to attosecond (10^−15^ to 10^−18^ s) timescale. This is achieved by using the lifetime of the core hole (created by the resonant excitation of an electron) as an internal clock.^19^

**Fig. 4:**
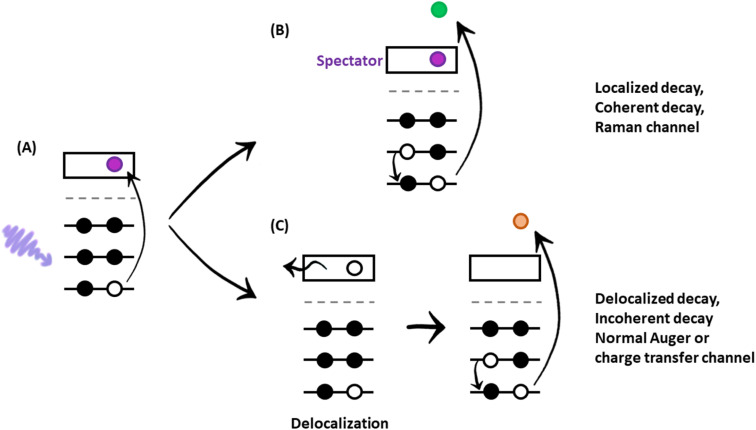
Illustration of important electronic processes in the core hole clock method. (A) Excitation of an electron from a core level (*e.g.* from the K-shell) to the conduction band by an X-ray with an energy around the absorption edge; (B) the excited electron in the conduction band (spectator) spectates the resonant Auger electron emission; (C) the excited electron leaves the conduction band (delocalizes) before the Auger electron is emitted.

**Fig. 5:**
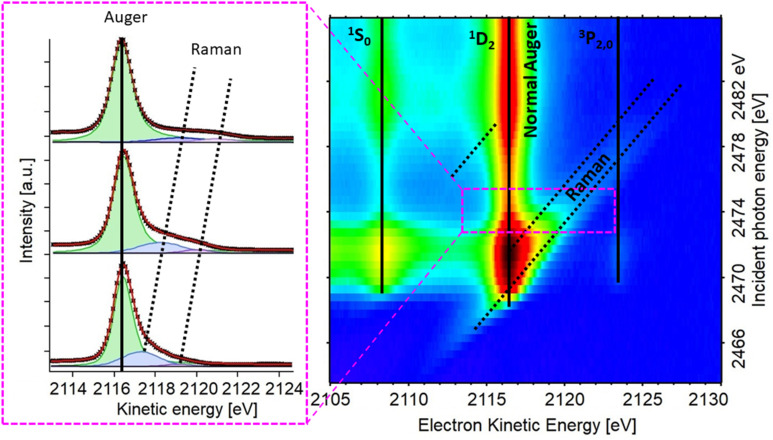
Resonant S KLL Auger map for the PbS reference thin film sample (right) and individual Auger spectra from the area marked with the pink rectangle (left).

The experiment begins with determining the absorption edge energy, in our case with XAS of the S K-edge. Around this photon energy is the region of interest for the resonant Auger study. Once an electron has been excited to the CB creating an excited state consisting of a core hole and an excited electron in the CB (Fig. 4A), the system can then relax through auto ionization and two different processes (channels) compete: spectator and charge transfer decays (Fig. 4B and C). We are thus ignoring the presumed small contribution of the radiative decay since sulfur is a light element. In literature, these channels are also referred to as coherent and incoherent decays^38^ or sometimes localized and delocalized decay,^39^ respectively. In spectator decay, the electron in the conduction band stays localized during the Auger decay and the emitted electron has a constant binding energy (*i.e.* a kinetic energy proportional to the incoming photon energy), this is also referred to as the Raman channel. In the other process, charge transfer occurs before the decay of the core hole yielding the same final state configuration as a normal Auger decay would (however with a photon energy below the ionization threshold) meaning that the emitted electron thereby has a constant kinetic energy. Scanning the Auger region over a range of photon energies around the resonance, we obtain a resonant Auger map. In this Auger map the charge transfer (incoherent) channels are seen as constant kinetic energy features (vertical solid lines, Fig. 5), while the spectator/Raman (coherent) channels are seen moving linearly to higher kinetic energy with the increase of excitation energy (dotted lines, Fig. 5). The intensities of the Raman contribution and the Auger contribution (integrated intensities of each channel, 
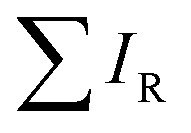
 and 
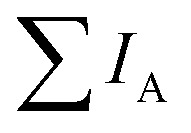
, respectively) therefore give the relative probabilities of the two processes (core hole decay without charge transfer and charge transfer followed by core hole decay), and their ratio is referred to as Raman ratio 
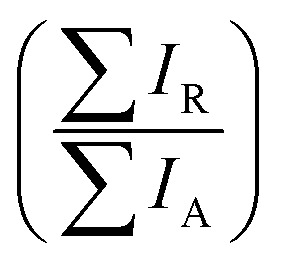
. One can consider these two processes as independent single exponential decays, where the decay time for the Raman channel is the core hole lifetime (*τ*_L_) and the decay time for the normal Auger channel is the charge transfer time (*τ*_CT_). The Raman ratio is then inversely proportional to the ratio of the decay times, and one can calculate the charge transfer (CT) times in our system according to:^19,40^2
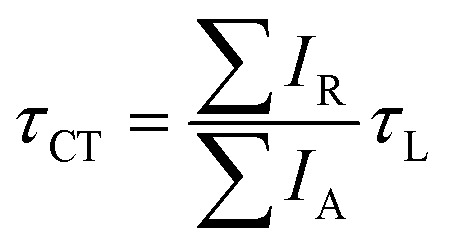


The determination of charge transfer times therefore depends on how accurately the Raman ratio can be determined and on the core hole lifetime, which is 1.27 fs for S 1s.^41^ In the case of our experiments, we would be able to determine charge transfer times in the range of tens of attoseconds to tens of femtoseconds.

The RAS map of the PbS ref sample recorded over the S K-edge resonance in the kinetic energy region of the S KLL Auger is shown in the Fig. 5, and RAS maps of all other PbS samples can be found in the ESI (Fig. S6 and S7†). We note that if the maps are integrated across the kinetic energy axis, the given spectra are the partial electron yield X-ray absorption spectrum similar to the XAS spectrum measured with a fluorescence detector (partial fluorescence yield).

**Fig. 6:**
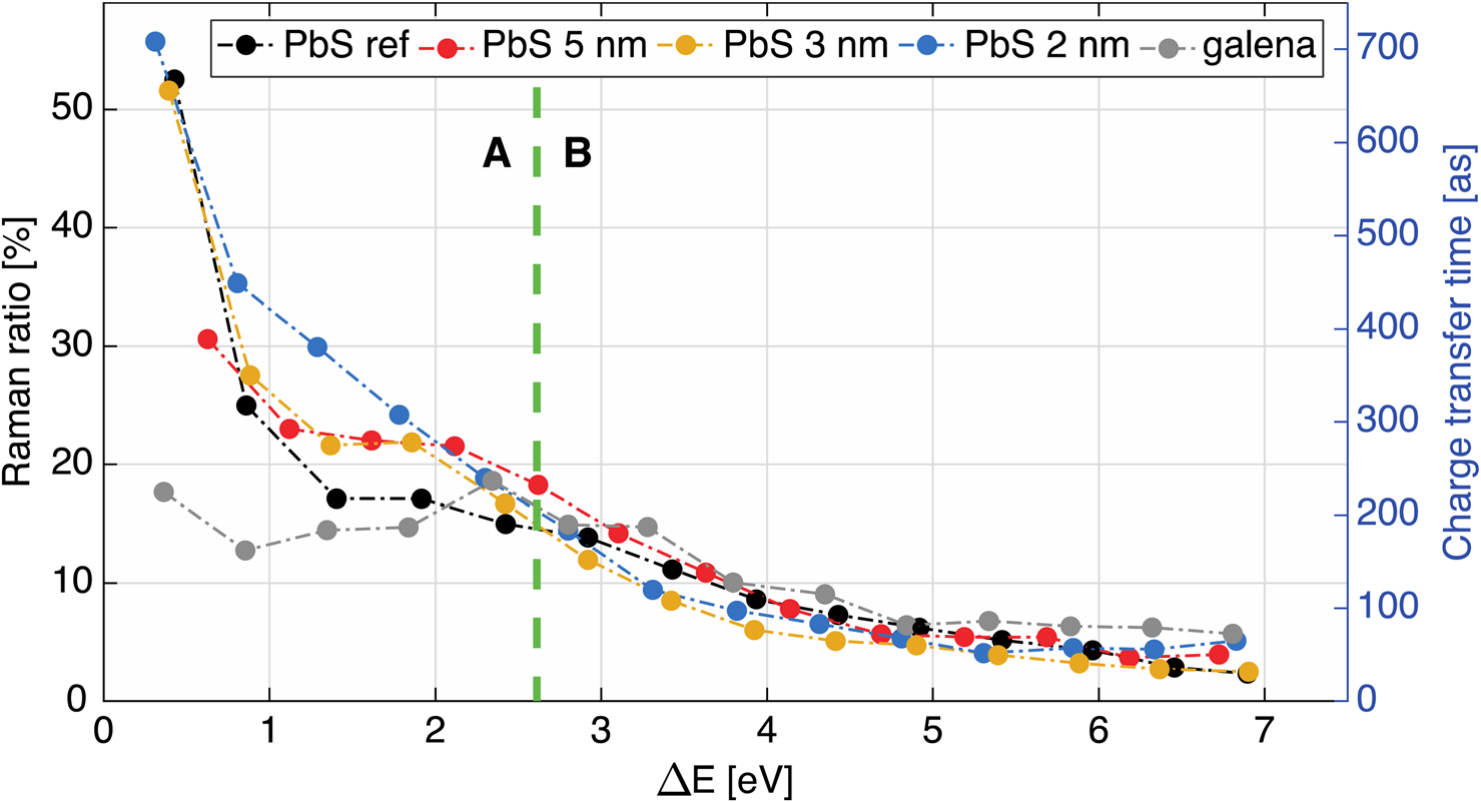
Raman ratio (left axis) and calculated charge transfer times in attoseconds (right axis) as a function of energy difference (Δ*E*) between the Raman feature at lower KE and the ^1^D_2_ Auger peak.

In the map in Fig. 5 we observe the most intense S KL_2,3_L_2,3_ Auger line (^1^D_2_ transition)^42^ along with two less intense channels (^1^S_0_, ^3^P_2,0_),^42^ marked with vertical lines, and focus in this paper is set on the most intense (main) Auger line. As described above, it is necessary to know the Raman ratio, which is given by intensity ratio of Raman and Auger feature from individual spectra of the resonant map, to determine the CT time. Therefore, the components of the spectra were manually deduced for each map, using the least squares curve fitting procedure described in the ESI.† The dispersive Raman features are marked with dotted diagonal lines in Fig. 5. The Raman feature in the PbS maps presented here is wide compared to the Raman feature in previously reported studies on sulfide compounds,^43–45^ In order to get a meaningful fit, the Raman contribution was assigned two Voigt peaks with controlled widths (Fig. 5, left). After several fitting iterations the calculated Raman ratio and CT times (see eqn (1)) were plotted against energy difference (Δ*E*) between the first Raman feature (at lower KE) and the ^1^D_2_ Auger peak (Fig. 6). For calculation of CT times the tabulated S 1s core hole lifetime with value of 1.27 fs was used,^41^ which allows our calculated times to be directly compared to times in the literature. From the overall shape of CT time data (Fig. 6) we may distinguish two regions, A and B. A comparison of typical individual spectra for the different samples in each region is shown in Fig. S10.† The Region B is the region where the electron is given a relatively higher excitation energy and the CT times are similar for all PbS samples. A similar trend of CT has been seen in a study of PCPDTBT:PCBM bulk heterojunctions, where CT is divided into intra- and inter-molecular CT.^23^ In analogous manner, we assign the region B to be dominated by interparticle CT, and region A by intraparticle CT. With the interparticle CT we refer to the electron hopping (or tunneling) from the excited QD to an adjacent QD, whereas with intraparticle CT we are referring to the electron movement or delocalization within the same QD. The CT time in interparticle CT region (B) is similar for all samples but appears a bit faster in smaller QDs. This suggests that the electrons hop between small QDs easier with higher excess energy, *i.e.* at higher excitation energy. Contrastingly, in part A, the electrons are given excitation energy just above resonance and in this part, there is a clear difference in charge transfer time between the different PbS samples.

**Fig. 7:**
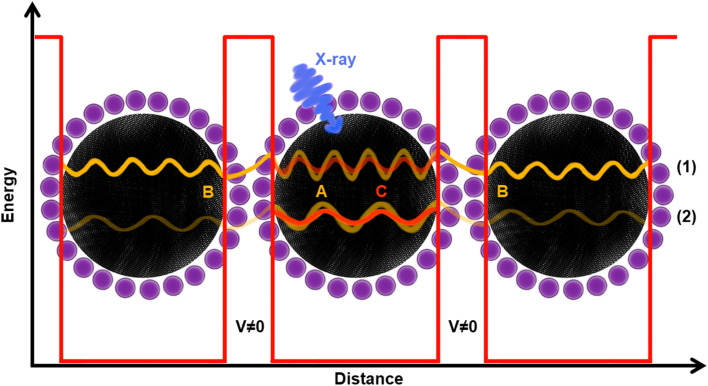
Graphical illustration of resonant charge transfer. The black spheres represent PbS quantum dots, while the purple ones represent iodide ions. The fading-yellow wave functions A in the middle dot represent two electrons being excited by the X-ray (blue arrow). Herein, two cases are presented: (1) when the electron ends up at a higher energy state as it is excited by a high energy X-ray (part B in Fig. 6), and (2) when an electron ends up at a lower energy state, as it is excited with a lower energy X-ray (part A in Fig. 6). Once excited, both electrons A have energy to tunnel away across the barrier (*V* ≠ 0 region) to a different dot (interparticle CT, yellow B wave functions) and/or travel to the potential barrier and be reflected of it back inside the particle (intraparticle CT, orange C wave function). However, in case of higher energy excitation, the probability of interparticle CT is higher than the intraparticle CT, while the opposite is true in case of lower excitation energy. In smaller dots, there would be less states to come back to (as the confinement effect is greater), which is manifested by longer CT (part A in Fig. 6).

Furthermore, a flat part of the CT time curve can be observed in region A with the CT time being constant for about 1 eV. This is most clearly seen for the 3 and 5 nm samples and also present, but not as clear, in the PbS ref sample. A similar CT time trend has previously been observed in a MoS_2_ – reduced graphene oxide interface, explained by the formation of Schottky barrier at the interface.^43^ However as our QD films are roughly 250 nm thickness, and the probing depth at these photon energies should not be more than 13 nm in the sample, we disregard influence of the substrate/PbS interface. Thus, one may conclude that the constant CT time region in Fig. 6 implies that there is a potential barrier which needs to be overcome before more states become accessible for charge transfer. This barrier could be formed by the ligand layer surrounding the quantum dots (illustrated in Fig. 7) and limit the probability of inter-particle CT at lower excitation energies. In this region, the differences in the probability of charge transfer might therefore come from differences in the probability of intraparticle transfer. The intraparticle CT in PbS 2 nm sample is slowest compared to other samples (roughly 400 as at 1.2 eV Δ*E*). This can be explained with a quantum mechanics model,^46^ where in potential wells with finite barrier height (analogous to quantum dots surrounded by ligands), the number of allowed energy states a wave function can have is dependent on the width of the potential well, *i.e.* quantum dot size. For instance, if a wave function (an electron) is given enough energy to be transmitted from one potential well to another one (from one dot to another) and on its way it encounters a potential barrier (a ligand layer) there are two possible outcomes: the electron wave function tunnels through the barrier (interparticle transfer), or is reflected from the barrier into the well (intraparticle CT), as illustrated in Fig. 7. The number of available bound states in the well (quantum dot) increases with increasing width of the well, meaning that the number of available states in a quantum dot depends on its size. In smaller dots, there are fewer available states which may overlap within one dot and allow for intraparticle CT or delocalization of electrons. For larger dots the density of overlapping states is larger with a corresponding increase in intraparticle delocalization probability and thus swifter charge transfer times in region A.

The reference samples show even shorter CT times (about 200 as at 1.2 eV Δ*E*) within this region, which indicates that CT is less limited at these energies. For the PbS ref thin film the charge transfer time does decrease with increasing energy, which indicates an increase in available states with increasing energy. This could be due to band bending towards the surface caused by the iodide treatment, which makes the surface more n-type compared to the surface of the galena crystal (Fig. S2†) or to grain boundaries in the polycrystalline PbS film that act as barriers for CT through the film. In the galena crystal the probability of delocalizing an electron inside the crystal does not seem to vary as much with excitation energy in region A in Fig. 6, which suggests that the number of available states in relatively constant with excitation energy. The values of CT times for PbS reported here are somewhat higher than CT times previously observed in polymers,^23^ but in range with values previously observed for metal-sulfides.^43,45^

## Conclusions

To summarize, we measured the ultrafast charge transfer times of quantum dot PbS, continuous PbS film and a natural crystal PbS material using Resonant Auger and core hole clock spectroscopy. Our results show that charge transfer in PbS QDs is slower at low excitation energies just above the resonance. In this region, charge transfer correlates with the diameter of the QDs and can be assigned to intraparticle CT. This can be qualitatively explained by smaller quantum dots having access to fewer states within the dot if considering the quantum dots as separated finite quantum wells with certain widths. However, if the excitation energy is above a certain threshold (which we assign to a potential barrier, present due to the ligands on the dots' surface), the charge transfer tends toward unity with increasing energy, *i.e.* the CT times shorten in all samples. This decrease is to some degree larger for the smallest QDs (PbS 2 nm and PbS 3 nm), which implies a faster CT time at higher excitation energies. Moreover, comparing the polycrystalline PbS film and the galena crystal we note that whereas the latter has a constant tunneling probability in region of lower excitation energies above resonance, the polycrystalline film has a sharp increase in tunneling probability (CT).

## Conflicts of interest

There are no conflicts to declare.

## Supplementary Material

RA-012-D2RA06091D-s001
