# Supplementary material for: Unravelling the ultrafast charge dynamics in PbS quantum dots through resonant Auger mapping of the sulfur K-edge
Source: RSC Adv. 2022 Nov 4;12(49):31671–9. doi: 10.1039/d2ra06091d (PMC9634717; doi:10.1039/d2ra06091d)
Supplement: RA-012-D2RA06091D-s001 [file RA-012-D2RA06091D-s001.pdf]

Electronic Supplementary Material (ESI) for RSC Advances.
This journal is © The Royal Society of Chemistry 2022

Supplementary information: Unravelling the ultrafast charge dynamics in PbS quantum dots through resonant Auger mapping of the sulfur K-edge

Tamara Sloboda,[1] Fredrik O. L. Johansson,[1,2,*] Birgit Kammlander,[1] Elin Berggren,[3] Sebastian Svanström,[3] Alberto García Fernández,[1] Andreas Lindblad,[3] Ute B. Cappel[1]

1 Division of Applied Physical Chemistry, Department of Chemistry, KTH Royal Institute of Technology, SE-100 44 Stockholm, Sweden

2 Sorbonne Université, CNRS, Institut des NanoSciences de Paris, INSP, F-75005, Paris, France

3 Division of X-ray Photon Science, Department of Physics and Astronomy, Uppsala University, Box 516, 751 20 Uppsala, Sweden

*fjson@kth.se

Sample fabrication:
Indium doped tin oxide (ITO) coated glass (1.1 mm ITO, ~12 Ω/sq) was used as substrate and cleaned with RBS™-25 concentrate, acetone, and ethanol, in that order, using an ultrasonic bath for 15 min and dried with air flow. Next, the ITO glass was treated with UV-ozone for 20 min directly before deposition of MgZnO nanoparticles, prepared and deposited as previously reported[1]. The INK PbS-PbI_2 QD films were prepared according to a previously reported method,[2] with slight modification: The 10 mg/ml PbS QD solution in octane was mixed in 1:1 vol. ratio with prepared ligand solution (0.12 M PbI_2 and 0.04 M ammonium acetate in dimethylformamide (DMF)). After vigorous mixing for 5 min the PbS QD are moved from octane to DMF phase which is visible by the color loss in octane layer, while the DMF layer becomes darkbrown. This marks the ligand exchange as complete. For the 5 nm dots this process was significantly slower and the fractions did not separate well immediately. That was improved with introduction of a small hot bath (hot tap water), which made the separation happen within minutes. Once the fractions were separated, the octane-OA phase was discarded and the OA residues are further removed with repeated washing with octane. After two washing steps, the toluene was added to precipitate the QDs, after which the solutions were centrifuged and the pellet was dried under vacuum for about 1 h. As a final step, the pellet was dispersed in butylamine:DMF (9:1 vol.) mixture to the concentration of 200 mg/ml. This concentrated ink was spin coated on top of the MgZnO layer as soon as possible as previously reported[1]. The samples were stored in darkness and inert atmosphere before measurement.

The samples used for the XRD measurements were prepared by drop-casting the PbS-PbI_2 ink solutions on a micro-slide glass and drying in 50-70 ^{o}C.

Material characterization:
The absorption and photoluminescence (UV-VIS-NIR) measurements were performed on Avantes AvaSpec-UV/Vis/NIR dual channel spectrometer. Tungsten halogen lamp (Avantes AvaLight HAL-S-Mini 10 W) was used as the excitation light source for absorption measurements and for calibrating the photoluminescence measurements. The excitation source for photoluminescence measurements was a 530 nm light source (Avantes AvaLight-HPLED-530 LED).

XRD was recorded using a PANalytical XPert PRO diffractometer with Cu Kα radiation at room temperature.

Additional Details Regarding RAS maps:
The Pb 4f core level were recorded in parallel to the Auger signal in order to note any sample degradation visible through formation of metallic Pb[1]. The Pb 4f core level remained stable for most of the samples, except for the PbS 2 nm, where a metallic lead Pb 4f signal accumulates over time (about 3 %, Figure S8). As this amount is not significantly larger than previously reported for the 3 nm PbS-PbI_2 QD sample[1], we do not expect this to have a significant effect on the results. However, it is the only sample in the series that formed the metallic lead.

All spectra from the resonant maps were analyzed using least squares cure fitting in IGOR using the SPANCF macros. The Auger peaks were fitted to a Doniach-Šunjić[3] spectral line shape, while the Raman peaks were fitted to a Voigt line shape. The spectral background was fitted to a Shirley function.[4] This fitting model was acquired through iterations by changing parameters when necessary. As the goal is to fit the areas of the dispersing and constant kinetic energy features over the whole excitation energy range we used two peaks for the Raman (dispersing) features. The normal Auger spectrum taken far above threshold provided positions for the constant kinetic energy signals.

The position of first and second Raman peaks were adjusted to the best fit (at a selected photon energy, e.g. 2475 eV, where the feature was still intense, but not significantly overlapping with Auger peaks), and after the fit was achieved, they were set to a constant BE distance to each other which was used in fitting all PbS resonant maps. Their absolute BE position was moved by the photon energy step in spectrum-by-spectrum fitting. The evolutions of the fitting parameters are plotted in the Supporting information (Figure S9). The small $^3P_{2,0}$ transition always had the same width and asymmetry parameters as the main $^1D_2$ transition.

Surface-volume ratio calculations
The surface to volume ratio was calculated according to:

$$\frac{surface}{volume}(QD) = \frac{A_{QD}}{V_{QD}} = \frac{4r_{QD}{}^2\pi}{{}^4/_3 r_{QD}{}^3\pi} = \frac{3}{r_{QD}} \quad (1)$$

where r_{QD} is the radius of QD.

The surface/bulk ratio was calculated as:

$$\frac{surface}{bulk} = \frac{V_{(iodide\ shell)}}{V_{QD}} = \frac{V_{(total)} - V_{QD}}{V_{QD}} \quad (2)$$

where the radius for $V_{(total)}$ is $r_{QD} + r_{I^-}$, where r_{I^-} is the ionic radius of I^-.

Supplementary Tables

Table S1 Fit parameters and calculated positions for exciton peak from absorption spectra, as well as the average size calculated from the width of three strongest peaks in XRD diffraction. The error of the size determined by XRD is the standard deviation of the calculation. The standard deviation of the absorption (σ_{ABS}) is calculated from the Gaussian fit of the exciton peak. The exciton peak position plus/minus σ_{ABS} is used to estimate the size distribution given in the main paper.

QD sample	Exciton peak position [eV]	Size from abs. spectrum [nm]	Size from XRD pattern [nm]	σ_{ABS} [eV]
**2 nm**	1.79	2.15	2.3±0.23	0.12
**3 nm**	1.29	3.14	2.8±0.11	0.05
**5 nm**	0.81	5.82	5.0±0.10	0.02

Table S2 PES core level (CL) positions and relative ratios of CL/Pb 4f for the PbS ref sample and QD samples. Spectra are energy calibrated to Au $4f_{7/2}$ at 84 eV

	**PbS ref**		**5 nm PbS**		**3 nm PbS**		**2 nm PbS**	
**CL**	BE [eV]	CL/Pb $4f_{tot}$	BE [eV]	CL/Pb $4f_{tot}$	BE [eV]	CL/Pb $4f_{tot}$	BE [eV]	CL/Pb $4f_{tot}$
**Pb 4f**	137.85	1	138.18	1	138.26	1	138.30	1
**S 2p**	161.04	0.51	161.31	0.41	161.28	0.32	161.30	0.31
**I 4d**	49.36	0.05	49.47	0.29	49.44	0.46	49.42	0.65
**VB_{onset}**	0.27	-	0.71	-	0.75	-	0.83	-
**VBM**	-	-	0.51	-	0.66	-	0.85	-

Supplementary Figures

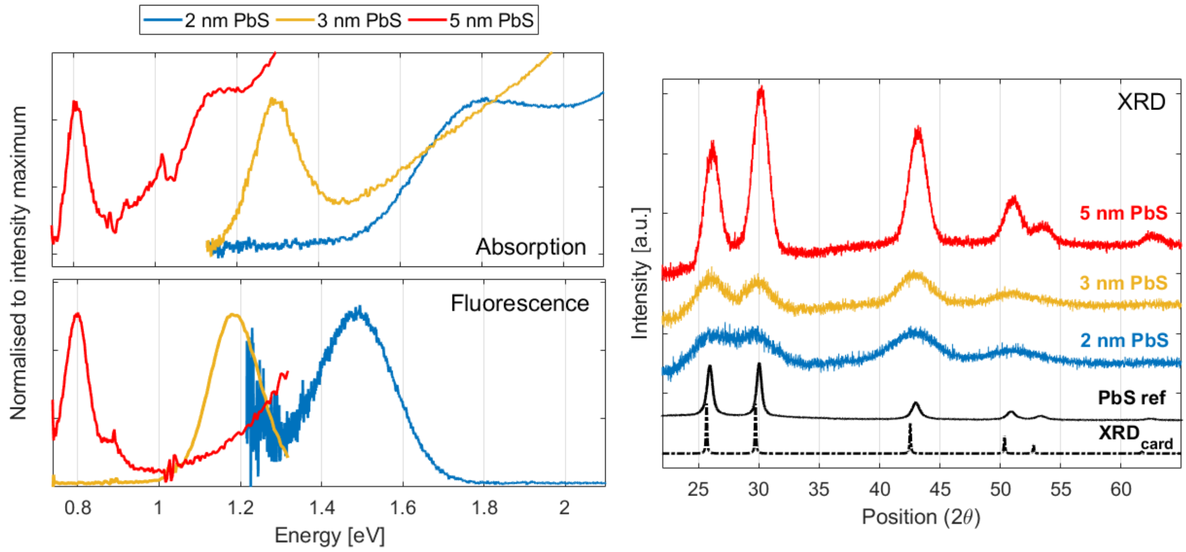

Figure S1 Absorption and photoluminescence spectra of PbS-OA of 3, 4 and 5 nm solutions in octane (left), normalized to the height intensity maximum of the exciton peak, and XRD diffractograms of theoretical PbS,[5] PbS thin film reference and PbS-OA particles of 2 nm, 3 nm and 5 nm (right)

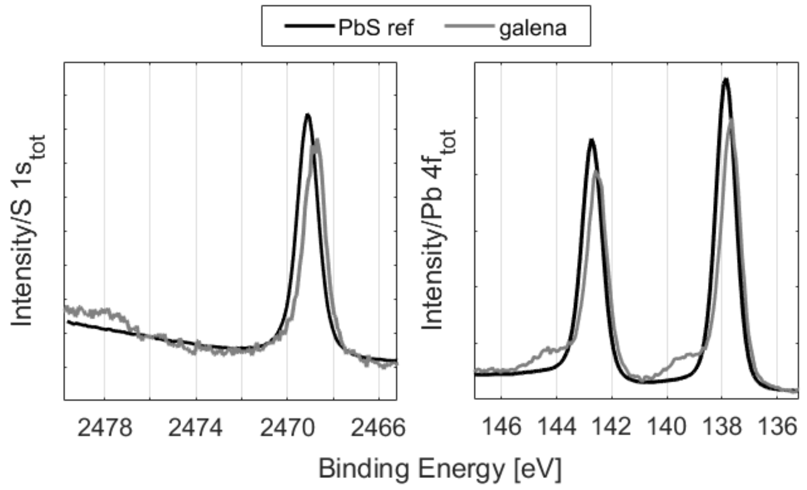

Figure S2. PES of PbS thin film with iodide surface treatment (PbS ref) and cleaved natural galena crystal (galena). Binding energy is calibrated to external Au reference.

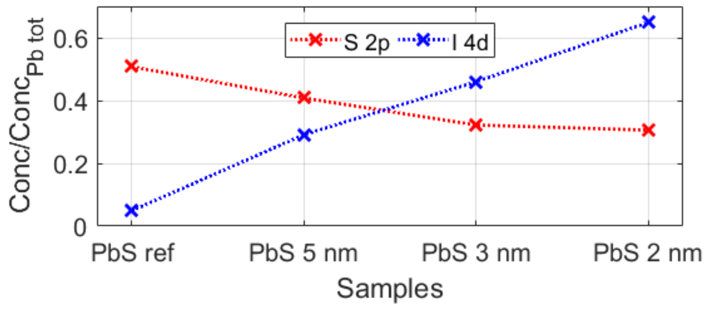

Figure S3. Content of S 2p (red) and I 4d (blue) in relation to total amount of Pb in differently sized PbS samples.

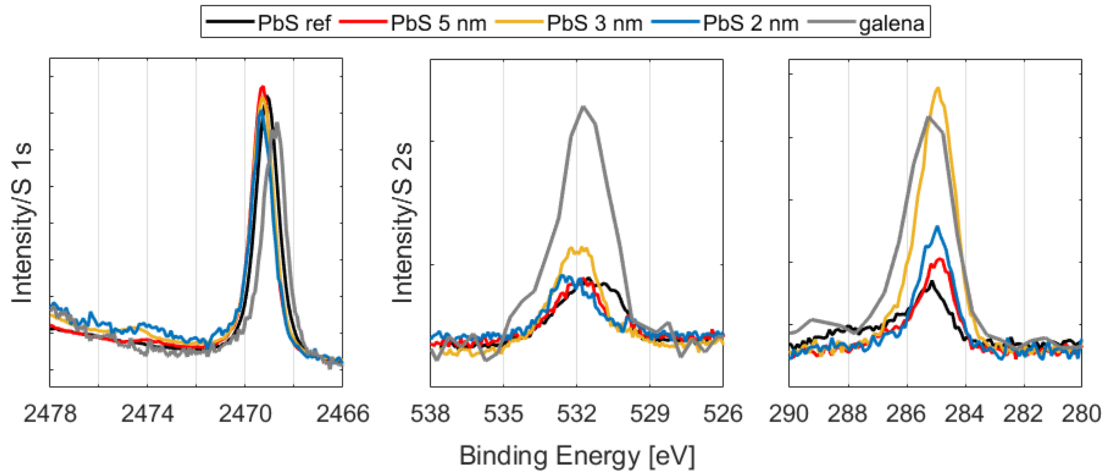

Figure S4. High resolution HAXPES spectra of the PbS samples: S1s, (left) O1s (middle), and C1s (right), calibrated against external gold reference mounted on the manipulator and normalized to S 1s (S 1s) and S 2s (O 1s and C 1s). Binding energy calibrated against external Au reference.

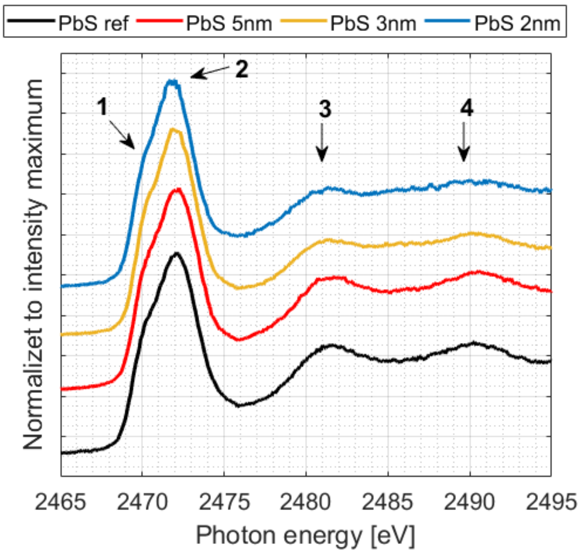

Figure S5. XAS spectra of PbS samples with highlighted main features which are explained in the main text. The photon energy is calibrated using Au gold foil mounted on the manipulator in the experiment chamber.

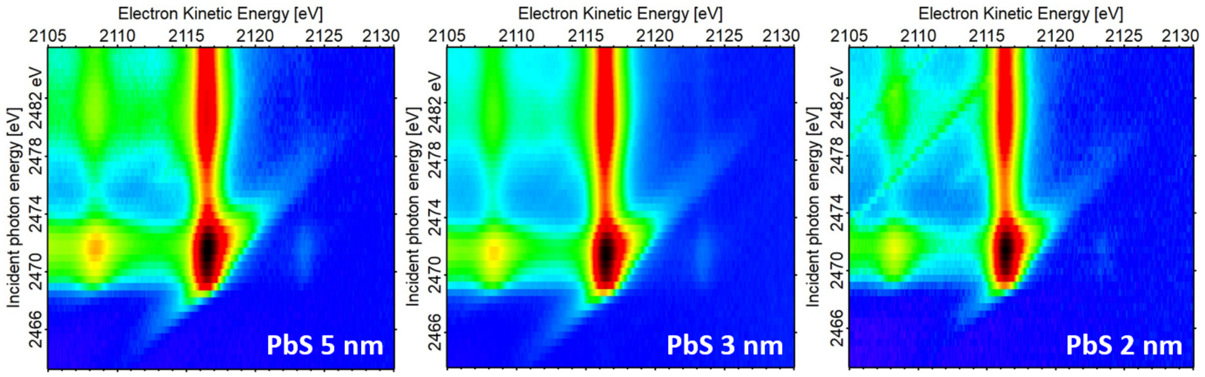

Figure S6. Resonant Auger maps of S KLL in PbS 5nm, PbS 3nm and PbS 2 nm quantum dot samples. Photon energy is calibrated using external Au reference.

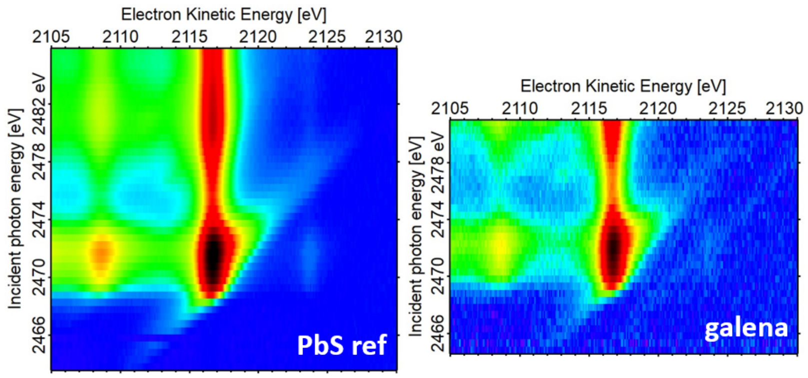

Figure S7. Resonant Auger maps of S KLL in thin film PbS reference (PbS ref) and galena crystal. Photon energy is calibrated using external Au reference.

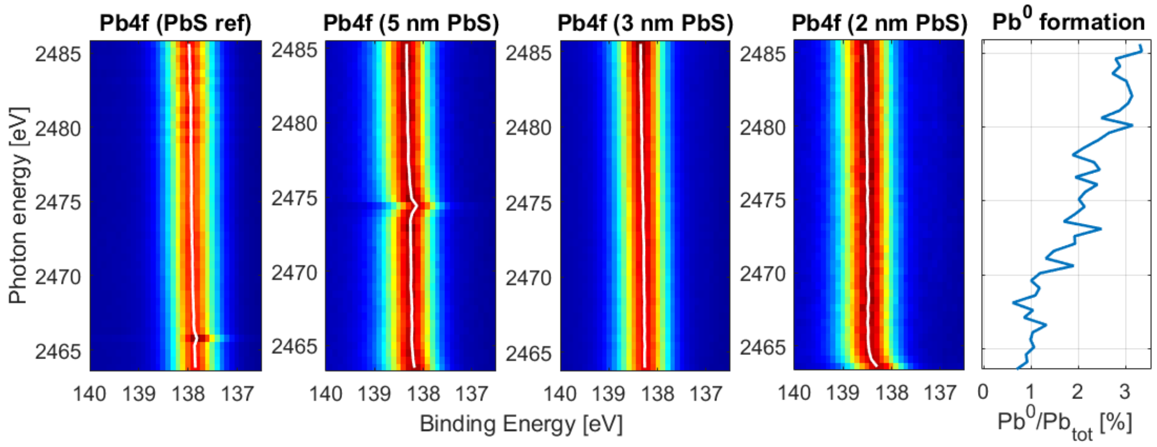

Figure S8. Position of Pb 4f core level during resonant Auger map measurements and the formation of metallic lead in 2 nm PbS sample. Calibrated to external Au reference.

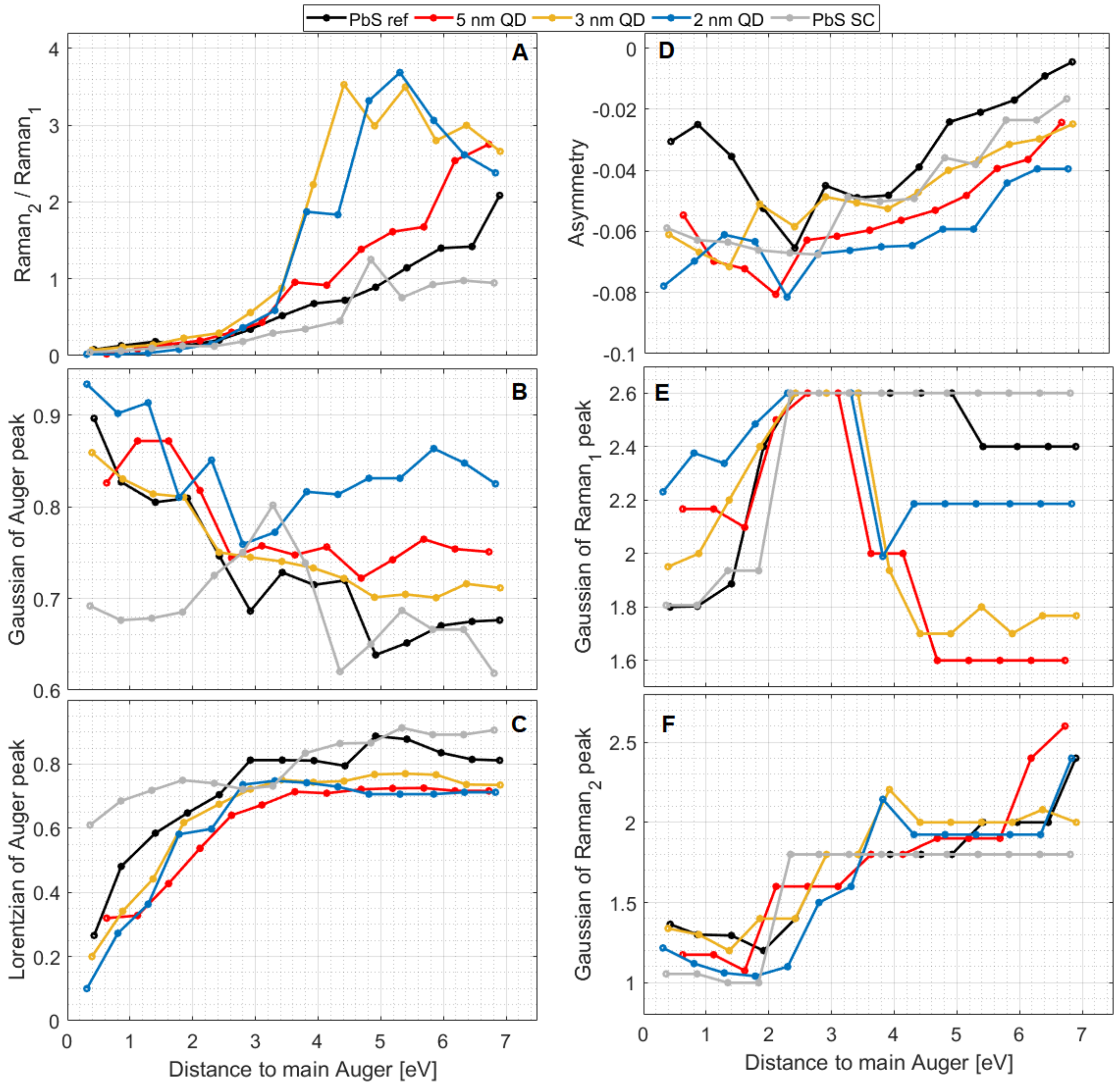

Figure S9. (A) Area ratio of two Raman feature, (B) Gaussian width of normal Auger line, (C) Lorentzian width of normal Auger line, (D) Asymmetry contribution in the normal Auger peak, (E) Gaussian width of the first Raman feature, (F) Gaussian width of the second Raman feature. Lorentzian of the Raman features was kept at 0.4 eV, and 0.3-0.2 eV in region when Raman features were less than 2 eV to the resonance

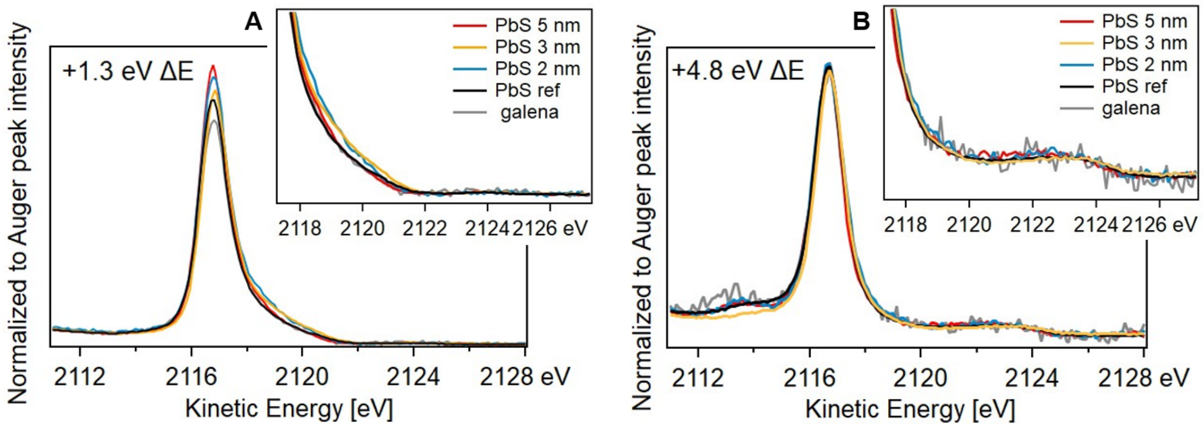

Figure S10. Individual spectra from the RAS maps of all PbS samples at the position about 1.3 eV ΔE (A) and at about 4.8 eV ΔE (B) (see Figure 6 in the main text)

References

1 T. Sloboda, S. Svanström, F. O. L. Johansson, E. Bryngelsson, A. Garcia-Fernandez, A. Lindblad and U. B. Cappel, *Phys. Chem. Chem. Phys.*, 2022, **24**, 12645.

2 A. Fischer, L. Rollny, J. Pan, G. H. Carey, S. M. Thon, S. Hoogland, O. Voznyy, D. Zhitomirsky, J. Y. Kim, O. M. Bakr and E. H. Sargent, *Adv. Mater.*, 2013, **25**, 5742–5749.

3 S. Doniach and M. Šunjić, *J. Phys. C Solid State Phys.*, 1970, **3**, 285.

4 D. A. Shirley, *Phys. Rev. B*, 1972, **5**, 4709–4714.

5 P. Villars and K. Cenzual, Eds., PbS Crystal Structure: Datasheet from ‘PAULING FILE Multinaries Edition - 2012’, https://materials.springer.com/isp/crystallographic/docs/sd_0531264.
